# ASSOCIATION BETWEEN SURGICAL VOLUME AND MORTALITY FROM COLON CANCER IN COLOMBIA: A NATIONAL COHORT

**DOI:** 10.1590/0102-6720202400041e1835

**Published:** 2024-12-02

**Authors:** María Fernanda CASTRO-CUARÁN, Edgar German JUNCA, Diego Felipe GONZALEZ-PATIÑO, Giancarlo BUITRAGO

**Affiliations:** 1 Universidad Nacional de Colombia, Faculty of Medicine, Department of Surgery - Bogotá (DC), Colômbia; 2 Hospital Universitario Nacional de Colombia, Department of Surgery, Bogotá (DC), Colômbia; 3 Universidad Nacional de Colombia, Faculty of Medicine, Clinical Research Institute, Bogotá (DC), Colômbia

**Keywords:** Colonic neoplasms, Colectomy, Survival, Mortality, Cohort studies., Neoplasias do colo, Colectomia, Sobrevida, Mortalidade, Estudos de coortes.

## Abstract

**BACKGROUND:**

Colon cancer is the third most common malignancy in Colombia, only exceeded by prostate and breast cancers. It is the second most common cancer among females and the third most common among males. The epidemiology of this disease has changed in Colombia, and its peak incidence has now surpassed that of gastric cancer.

**AIMS:**

We aimed to determine the association between hospital surgical volume and mortality in patients with colon cancer undergoing surgical resection in Colombia.

**METHODS:**

This was a national retrospective cohort study based on administrative data and included adult patients undergoing surgical resection for colon cancer who were enrolled in Colombia’s contributory health system between 2012 and 2017. We defined exposure as the hospital’s surgical volume where the colon cancer surgery was performed. We classified the patients as exposed to a high surgical volume (above the 90^th^ percentile of the provider distribution) and a low surgical volume (under the 90^th^ percentile). The main outcomes were 30-day and 1-year mortality. Multivariate Poisson regressions were used to identify the association between exposure and mortality rates.

**RESULTS:**

The study included 4,647 patients, of which 4,188 underwent surgery at hospitals with a colectomy volume lower than 33 per year and 459 underwent surgery at institutions with volumes equal to or higher than 33 per year. In the multivariate analysis, after adjusting for observable variables, a lower risk of 30-day mortality was found in patients who underwent surgery at high surgical volume institutions (relative risk - RR 0.57, 95% confidence interval - 95%CI 0.033-0.97). No differences were found in the one-year mortality.

**CONCLUSION:**

The high surgical volume of a hospital is associated with a 30-day mortality in colon cancer, as described in other studies, but the 1-year mortality did not show this association. Prospective studies are required to establish a causal relationship.

## RESUMO

**INTRODUÇÃO:**

O câncer de cólon é a terceira doença maligna mais comum na Colômbia, superado apenas pelos cânceres de próstata e de mama. É o segundo câncer mais comum entre as mulheres e o terceiro mais comum entre os homens. A epidemiologia desta doença mudou na Colômbia, e o seu pico de incidência já ultrapassou a do câncer gástrico.

**OBJETIVOS:**

Determinar a associação entre o volume cirúrgico do Hospital e a mortalidade em pacientes com câncer de cólon submetidos a ressecção cirúrgica.

**MÉTODOS:**

Estudo de coorte retrospectivo nacional baseado em dados administrativos e incluiu pacientes adultos submetidos a ressecção cirúrgica para câncer de cólon que estavam inscritos no sistema de saúde contributivo entre 2012 e 2017. Definimos a exposição como o volume cirúrgico do hospital onde a cirurgia de câncer de cólon foi realizada. Classificamos os pacientes como expostos a um alto volume cirúrgico e expostos a um baixo volume cirúrgico. Os principais resultados foram a mortalidade em 30 dias e em 1 ano. Regressões de Poisson multivariadas foram utilizadas para identificar a associação entre a exposição e as taxas de mortalidade.

**RESULTADOS:**

Foram incluídos 4.647 pacientes, dos quais 4.188 pacientes foram submetidos a cirurgia em hospitais com um volume de colectomia inferior a 33 por ano e 459 foram submetidos a cirurgia em instituições com volumes iguais ou superiores a 33 por ano. Na análise multivariada, após o ajuste para variáveis observáveis, foi encontrada uma menor taxa de mortalidade em 30 dias em pacientes submetidos a cirurgia em instituições com alto volume cirúrgico. Não foram encontradas diferenças na mortalidade em um ano.

**CONCLUSÕES:**

O alto volume cirúrgico de um hospital está associado à mortalidade em 30 dias no câncer de cólon, como descrito em outros estudos, mas a mortalidade em 1 ano não mostrou essa associação. Estudos prospectivos são necessários para estabelecer uma relação causal.

## INTRODUCTION

According to data from GLOBOCAN 2020, colon cancer is the fourth most common malignancy worldwide, with an incidence rate of 19.5 per 100 thousand inhabitants^20^. In Colombia, colon cancer is the third most common malignancy, with an incidence rate of 16.9 per 100 thousand inhabitants and is only exceeded by prostate and breast cancers. Colon cancer has a mortality rate of 8.2 per 100 thousand inhabitants, which ranks it the fifth most common cause of cancer-related mortality. Colon cancer is the second most common cancer among females and the third most common among males. The epidemiology of this disease has changed in Colombia, and its peak incidence has now surpassed that of gastric cancer^5,12^. A study from Cali, Colombia, reported an increase in the incidence rate from 4 per 100 thousand (1962-1966) to 13 per 100 thousand (2003-2007). A similar trend was observed in mortality, which has increased from 3.1 per 100 thousand (1984-1988) to 9.3 per 100 thousand (2009-2012)^7^. Another survey from Bucaramanga, Colombia, reported a rise in colorectal cancer incidence from 13.3 cases per 100 thousand males and 12.2 cases per 100 thousand females (2003-2007) to 14.3 and 13.5, respectively (2008-2012)^21^.

This incidence is attributed to changes in lifestyle, such as diet, sedentary behavior and being overweight. It is known that diets high in red meat (relative risk - RR 1.21; 95% confidence interval - 95%CI 1.13-1.29) and processed meat (RR 1.19 95%CI 1.12-1.27), alcohol (RR 1.56 95%CI 1.42-1.70), smoking (RR 1.16 95%CI 1.09-1.24), obesity (RR 1.19 95%CI 1.11-1.29), and inadequate physical activity are risk factors for this malignancy^2,12^. Developed countries exhibit decreased mortality that is primarily associated with the introduction of colonoscopy screening programs^3,18^, which are recommended for patients who are at least 45 or 50 years of age depending on the risk group^16,24^. In Colombia, 50% of the health services are located in Bogota, Antioquia y Valle del Cauca, and 87.9% of the service supply is in the private sector^4^. The departments with the highest incidence and mortality due to colorectal cancer are Quindío, Bogota, Risaralda, Caldas and Valle del Cauca^17^.

Surgical therapy and systemic adjuvant chemotherapy remain the cornerstones of colon cancer treatment at certain disease stages^2,23^. The prognostic determinants are related to the patient (e.g., age, sex, comorbidity, background) and the biology of the tumor (e.g., histopathology, stage, tumor differentiation)^10^; however, surgery-related factors, such as complete mesocolic excision with high ligation and lymphadenectomy, also play an important role. This technique is associated with disease-free survival with reduced relative risks of 40, 28 and 33% at the first, third and fifth years after surgery, respectively^8^. Furthermore, the surgeon’s qualification has been demonstrated to be important, as shown by a UK study which reported lower postoperative mortality when the procedure was performed by a specialist in colorectal surgery compared with a general surgeon (4.5 vs. 7%, p=0.032)^16,19^.

To the best of our knowledge, no information on the hospital’s surgical volume of patients undergoing colon resection from low-income countries has been described. Considering this context, this study intends to determine the association between surgical volume and mortality in patients with colon cancer undergoing surgical resection in Colombia, a country with both lowand middle-income regions.

## METHODS

### Study type and population

This was a retrospective cohort study of adult patients undergoing surgical resection for colon cancer in the contributory health regimen in Colombia from 2012 to 2017. All patients older than 18 years with a colon cancer diagnosis based on the International Classification of Diseases 10^th^ Revision (ICD-10 classification) and who underwent surgical resection covered by any of the ten health insurers (reporting complete data to the Ministry of Health Universal Product Code - UPC sufficiency database) were included. Patients who withdrew from the contributory health regimen within 90 days after colectomy were excluded. Mortality information was obtained from death certificates from the Unique Enrollment Registry (*Registro Unico de Afiliados* - RUAF). The UPC database contains information that Colombia’s health system insurers send to the Ministry of Health and is used to estimate the premiums that the system recognizes for each person enrolled in the health system. This database is highly standardized and contains detailed information about all services used by the enrollees, including the type of service provided, ICD-10 codes, date of service, municipality, sex, age, insurer, service provider and the cost paid by each insurer (the cost incurred by the health system for each enrollee). The RUAF death certificate database contains information on all deaths in the country and includes the date and cause of death. Death certificates in Colombia have a coverage of over 90%. The databases were anonymized, and the study was approved by the Ethics Committee of the School of Medicine at the Universidad Nacional de Colombia (number 020-230 2020).

### Exposure, outcomes and control variables

The primary exposure variable was the average annual volume of colectomies performed at the hospital at which the patient underwent colon resection. We divided the sample into an exposed cohort, which included patients who were treated at hospitals with high surgical volume (hospital in 90^th^ percentile or higher), and an unexposed cohort, which included patients who were treated at hospitals with low surgical volume (hospital under the 90^th^ percentile). The main outcomes were the 30-day and 1-year mortality rates after surgery. We included age, sex, type of chemotherapy, Charlson comorbidity index (CCI) (including metastatic disease), geographical region, and health insurer as variable controls.

### Statistical analysis

All baseline characteristics and outcomes were described using absolute and relative frequencies for categorical variables and means and standard deviations for continuous variables. We estimated crude and adjusted relative risks for 30-day and 1-year mortality for highand low-volume surgical exposure using Poisson regressions. To obtain the adjusted relative risks, multivariate Poisson models were used to control for age, sex, CCI, type of chemotherapy, geographic region, and health insurer. The 95% CIs were estimated. All analyses were performed with Stata 17 MP (Universidad Nacional de Colombia license).

## RESULTS

The study included 4,647 patients with colon cancer who received surgical management in Colombia in the contributory regime between 2012 and 2017. The 90^th^ percentile was 33 surgeries per year. A total of 4,188 patients underwent surgery at hospitals with a colectomy volume lower than 33 per year and 459 underwent surgery at institutions with volumes equal to or higher than 33 per year. Overall, 53.13% in the low surgical volume cohort were female and 51.80% in the high surgical volume cohort were female. Most of the patients were in the 60-79-year age group, and no significant differences were observed between the cohorts. According to region, 83.44% of patients underwent surgery at institutions with high surgical volumes in Bogota, and only 2.18 and 12.85% underwent surgery in central and eastern regions, respectively. Most patients had a CCI of 2, and no differences were observed between the groups. A higher number of patients received adjuvant chemotherapy or no chemotherapy, and no significant differences were seen among the groups (Table 1).

**Table 1:** Baseline characteristics of cohorts.

	High surgical volume (n=4,188)	Low surgical volume (n=459)	p-value
Sex (%)			
Female	2,225 (53.13)	238 (51.85)	0.603
Male	1,963 (46.87)	221 (48.15)	
Age category in years; n (%)			
<40	256 (6.11)	14 (13.05)	0.92
40-59	1,282 (30.61)	118 (25.71)	
60-79	2,122 (50.67)	258 (56.21)	
>79	528 (12.61)	69 (15.03)	
CCI; n (%)			
2	2,341 (55.9)	255 (55.6)	0.990
3-5	1504 (35.91)	166 (36.17)	
>5	343 (8.19)	38 (8.28)	
Type of chemotherapy; n (%)			
None	1,784 (42.6)	208 (45.32)	0.499
Neoadjuvant	357 (8.52)	35 (7.63)	
Adjuvant	2,047 (48.88)	216 (47.06)	
Geographical region; n (%)			
Atlantic	462 (11.03)	0 (0%)	0.000
Bogota DC	1,105 (26,38)	383 (83.44)	
Central	1,395 (33.31)	10 (2.18)	
Oriental	679 (16.21)	59 (12.85)	
Pacific	528 (12.61)	1 (0.22)	
Other departments	19 (0.45)	6 (1.31)	
Other Health insurers; n (%)			
1	1,393 (33.26)	302 (65.8)	0.000
2	467 (11.15)	2 (0.44)	
3	442 (10.55)	0 (0)	
4	469 (11.2)	52 (11.33)	
5	347 (8.29)	2 (0.44)	
6	392 (9.36)	25 (5.45)	
7	678 (16.19)	76 (16.56)	

CCI: Charlson Comorbidity Index.

The bivariate analysis reported a 30-day mortality of 1.75 deaths per thousand surgeries in the low surgical volume group and 1.337 in the high surgical volume group; the RR was 0.76 for the high surgical volume group, and no statistically significant differences were observed. Moreover, no association was found between the 30-day mortality and female sex, age or geographic region. However, a lower 30-day mortality was seen in patients undergoing neoadjuvant chemotherapy (RR 0.62, 95%CI 0.42-0.95), and patients with a CCI of 3-5 and >5 had an increased risk of death compared with those with a CCI=2 (Table 2). Regarding 1-year mortality, rates of 6.44 and 6.66 deaths per thousand surgeries were observed in the low-volume and high-volume groups, respectively, but no statistically significant differences were found. A higher risk of 1-year mortality was seen in the 60-79-year age group and in those older than 79, as well as in patients with a higher CCI. Treatment with adjuvant chemotherapy was associated with a lower risk of death. No associations were found between sex and geographic region (Table 2).

**Table 2:** Bivariate analysis between 30-day and 1-year mortality and characteristics of sample.

	30-day mortality			1-year mortality		
	RR	95%CI	p-value	RR	95%CI	p-value
Hospital volume						
Low surgical volume	Ref.					
High surgical volume	0.76	0.47-1.23	0.266	1.03	0.83-1.29	0.74
Age category in years						
<40	4.02-4.07	0	0.972	0.52	0.36-0.76	0.01
40-59	0.400	2.15-3.77	0.00	0.63	0.53-0.74	0.000
60-79	Ref.					
>79	2.8	2.15-3.77	0.00	1.79	1.51-2.11	0.000
Sex						
Female	Ref.					
Male	0.99	0.770-1.291	0.986	0.99	0.870-1.134	0.923
CCI						
2	Ref.					
3-5	1.60	1.219-2.116	0.001	1.31	1.142-1.514	0.000
>5	2.08	1.385-3.132	0.000	1.55	1.242-0.06	0.000
Type of chemotherapy						
None	Ref.					
Neoadjuvant	0.62	0.418-0.946	0.026	1.01	0.825-1.246	0.893
Adjuvant	6.14-6.09	0	0.981	0.35	0.304-0.414	0.000
Geographical region						
Atlantic	Ref.					
Bogota DC	1.57	0.902-2.733	0.111	1.01	0.797-1.290	0.907
Central	1.71	0.984-2.976	0.057	1.00	0.784-1.275	0.997
Oriental	1.60	0.8812.912	0.122	1.06	0.813-1.383	0.661
Pacific	1.53	0.810-2.888	0.190	0.99	0.744-1.327	0.969
Other departments	1.25	0.166-9.519	0.824	0.43	0.106-1.761	0.243

RR: risk ratio; 95%CI: confidence interval (Crude Risk Ratios were estimated using Poisson regressions); CCI: Charlson Comorbidity Index.

The multivariate analysis showed a decreased risk of 30-day mortality in patients who underwent surgery at high surgical volume institutions (RR 0.57; 95%CI 0.033-0.97). However, for the 1-year mortality, no differences were seen among the cohorts after adjusting for all the variables (RR 0.95; 95%CI 0.74-1.22) (Table 3). Adjusted risk ratios were estimated using Poisson regressions controlling by sex, age, CCI, type of chemotherapy, health insurer and geographical region.

**Table 3:** Association between hospital surgical volume and 30-day and 1-year mortality.

	30-day mortality			1-year mortality		
	aRR	95%CI	p-value	aRR	95%CI	p-value
Hospital volume						
Low surgical volume	Ref.					
High surgical volume	0.55	0.33-0.97	0.042	0.95	0.74-1.22	0.7

aRR: adjusted Risk Ratio; 95%CI: confidence interval.

## DISCUSSION

This national cohort from a middle-income country, which was analyzed using administrative databases, shows that patients with colon cancer who undergo colectomies at hospitals with a high surgical volume (33 or more surgeries per year) have a lower mortality rate at 30 days, but no differences were observed in the mortality rate at one year. To our knowledge, this is the first study of its kind in low-resource settings. In the management of malignancies, there is a tendency to centralize treatment at high-volume centers with specialized professionals who have the most experience due to the assumption that these patients will have better outcomes compared with patients treated at other centers. In addition, high-volume centers have better technological resources, multidisciplinary teams and intensive care units. These differences are especially intricate in our country given the socioeconomic, demographic and geographic conditions, which result in malignant pathology being treated in many scenarios by general surgeons at hospitals with high heterogeneity.

This study found that a greater portion of patients who underwent surgery at high-volume hospitals were in Bogota, which reflects the centralization of cancer treatment in Colombia. Likewise, in regions such as the Atlantic and Pacific, no patients underwent surgery at high-volume centers, which demonstrates the geographical inequity in access to specialized treatment in this country. In Colombia, 50% of the health services for cancer are located in Bogota, Antioquia y Valle^4^, which should be considered when studying the factors associated with disease survival, as this concept presupposes that rural and remote areas have lower access to screening and advanced care. A systematic review in Australia reported lower survival in nonmetropolitan areas as well as differences in the clinical management of colorectal cancer patients; nonetheless, these differences did not seem to be associated with laborious access to therapy rather than individual patient characteristics and regional characteristics, although evidence is still scarce^13^. In our experience, no differences in mortality were observed among various regions, but some differences do exist in access to high surgical volume hospitals.

It is necessary to emphasize the importance of the appropriate use of adjuvant chemotherapy since this treatment can have a positive impact on the survival of patients at high-risk stages, especially if treatment is initiated before the sixth postoperative week^23^. In this study, 48.6% of patients received adjuvant chemotherapy, which implies some form of advanced disease; however, given the limited data, we could not establish cancer staging in this population. Accordingly, a Canadian study found that in patients with stage III colorectal cancer with no referral to oncology, 38% had a more than 50% likelihood of receiving adjuvant chemotherapy if they had been referred^6^. Other factors that limit access to chemotherapy include poverty, low income, lack of insurance, lack of primary care physicians and higher tier hospitals^9^.

According to the multivariate analysis, patients who underwent surgery at high surgical volume institutions had a lower risk of 30-day mortality; this result is in agreement with other studies, such as the meta-analysis by Huo et al., which describes the association between high surgical volume hospitals and high-volume surgeons with reduced 30-day mortality (hazard risk - HR 0.83; 95%CI 0.78-0.87, p<0.001 & HR 0.84; 95%CI 0.80-0.89, p<0.001) and reduced intraoperative mortality (HR 0.82; 95%CI 0.76-0.86, p<0.001 & HR 0.50; 95%CI 0.40-0.62, p<0.001). Similarly, high-volume surgeons are associated with a higher survival rate at five years, higher lymphadenectomy and lower rates of recurrence, operation time, hospital stay and costs^11^. The highest volume hospitals and surgeons had the best results, yet this relationship is not linear because other factors might establish/alter these results. It must be emphasized that no clear limits exist between highand low-volume institutions because each study uses different cutoff values^11^. Other meta-analyses have reported similar conclusions^14,15,22^.

In terms of the 1-year mortality outcome, the crude mortality rate was higher in the low-volume group, and statistically significant differences were observed in the bivariate analysis, notwithstanding that this association was dismissed after the multivariate adjustment. Archampong et al. described a higher total survival at five years in colorectal cancer patients treated at high-volume hospitals (HR 0.90, 95%CI 0.85-0.96) by high-volume surgeons (HR 0.88, 95%CI 0.83-0.93) and colorectal specialists (HR 0.81, 95%CI 0.71-0.94). Operative mortality was lower when the surgery was performed by high-volume surgeons (OR 0.77, 95%CI 0.66-0.91) and specialists (OR 0.74, 95%CI 0.60-0.91), but no association with high-volume centers was observed (OR 0.93, 95%CI 0.84-1.04)^1^. On the contrary, an association was found between high-volume hospitals and lower operative mortality, as described in studies from the USA, which suggests variability in health providers among countries; this demonstrates the importance of conducting studies such as these in each country and health care system. These results must be carefully interpreted since other variables may act as confounding factors, including patient-related (age, sex, socioeconomic status, comorbidity, tumor biology, staging) and hospital-related (other specialty assistance, surgical philosophy, nurse patient ratio) factors^14^.

The main limitations of this study are its retrospective nature, and given that data were obtained from administrative databases, not enough clinical information is presented to identify all possible confounding factors. Moreover, no data were obtained on the subsidiary health regimen. Nevertheless, this study provides important results due to its large sample size and national representation.

The Colombian health care system has one major flaw related to limited access to cancer therapy at specialized centers, since only 9.8% received treatment at high surgical volume centers, and 83% of these patients were managed in Bogota, thus demonstrating persisting centralization of specialized services.

## CONCLUSIONS

These study findings suggest a higher 30-day mortality for patients who undergo surgery at high-volume centers, which contrasts with the 1-year mortality in which no differences were reported. This is the first study in Colombia to establish an association between surgical volume and mortality in patients with colon cancer, and despite limitations due to its retrospective design and limited data on oncologic variables (staging), the national basis of this study means that it provides valuable information that might constitute the foundations of future prospective studies.
